# The Relative Contribution of NMDARs to Excitatory Postsynaptic Currents is Controlled by Ca^2+^-Induced Inactivation

**DOI:** 10.3389/fncel.2016.00012

**Published:** 2016-01-29

**Authors:** Fliza Valiullina, Yulia Zakharova, Marat Mukhtarov, Andreas Draguhn, Nail Burnashev, Andrei Rozov

**Affiliations:** ^1^OpenLab of Neurobiology, Kazan Federal UniversityKazan, Russia; ^2^Department of Physiology and Pathophysiology, University of HeidelbergHeidelberg, Germany; ^3^INMED, Institut de Neurobiologie de la Méditerranée UMR901, Aix-Marseille UniversitéMarseille, France; ^4^INSERM U901Marseille, France

**Keywords:** excitation, NMDA receptor, calcium, modulation, action potentials, firing properties

## Abstract

NMDA receptors (NMDARs) are important mediators of excitatory synaptic transmission and plasticity. A hallmark of these channels is their high permeability to Ca^2+^. At the same time, they are themselves inhibited by the elevation of intracellular Ca^2+^ concentration. It is unclear however, whether the Ca^2+^ entry associated with single NMDAR mediated synaptic events is sufficient to self-inhibit their activation. Such auto-regulation would have important effects on the dynamics of synaptic excitation in several central neuronal networks. Therefore, we studied NMDAR-mediated synaptic currents in mouse hippocampal CA1 pyramidal neurons. Postsynaptic responses to subthreshold Schaffer collateral stimulation depended strongly on the absence or presence of intracellular Ca^2+^ buffers. Loading of pyramidal cells with exogenous Ca^2+^ buffers increased the amplitude and decay time of NMDAR mediated EPSCs (EPSPs) and prolonged the time window for action potential (AP) generation. Our data indicate that the Ca^2+^ influx mediated by unitary synaptic events is sufficient to produce detectable self-inhibition of NMDARs even at a physiological Mg^2+^ concentration. Therefore, the contribution of NMDARs to synaptic excitation is strongly controlled by both previous synaptic activity as well as by the Ca^2+^ buffer capacity of postsynaptic neurons.

## Introduction

In the mammalian central nervous system, excitatory synaptic transmission is mediated by glutamate which co-activates postsynaptic NMDA and AMPA receptors (AMPAR and NMDAR). Fast synaptic currents are mediated by AMPAR channels, whereas NMDAR channels generate slower and longer lasting currents (Forsythe and Westbrook, 1988; Bekkers and Stevens, 1989; Stern et al., 1992; Spruston et al., 1995). NMDARs are highly permeable to Ca^2+^ (Ascher and Nowak, 1988) and act as gatekeepers for the Ca^2+^ influx into dendritic spines during synaptic activity (Perkel et al., 1993; Malinow et al., 1994). NMDAR function can be modulated by a large number of extracellular agents, including Mg^2+^, glycine, Zn^2+^, polyamines and protons (Collingridge and Lester, 1989; Hollmann and Heinemann, 1994), as well as by the intracellular activities of protein kinases and protein phosphatases (Kotecha and MacDonald, 2003). In addition, numerous studies have shown that an increase in intracellular calcium concentration ([Ca^2+^]_i_) causes a reversible reduction of NMDA-activated currents, irrespective of the source of calcium (Legendre et al., 1993; Vyklicky, 1993; Medina et al., 1994; Kyrozis et al., 1995; Umemiya et al., 2001). The mechanism of Ca^2+^ induced inactivation of NMDARs (CIIN) involves calmodulin binding to the C-terminal of the GluN1 subunit and a subsequent reduction in the channel’s open probability (Ehlers et al., 1996; Zhang et al., 1998). The functional consequences of CIIN, however, are not well understood. If calcium entry via NMDAR suffices to suppress further activation of the channels, it could mediate an important negative feedback regulation for synaptic excitation under physiological conditions (Rosenmund et al., 1995). This question has not been directly addressed in the past, because most NMDAR-mediated responses were recorded at low extracellular Mg^2+^ concentration, with prolonged (≫ 1 ms) agonist application and without exact estimates of NMDA-mediated Ca^2+^ influx. However, even at resting membrane potentials (≃−70 mV) and in the presence of physiological Mg^2+^ concentrations NMDARs still act as the main synaptic source of Ca^2+^ entry (Kovalchuk et al., 2000), suggesting the possibility that CIIN shapes the postsynaptic Ca^2+^ dynamics and the kinetics of excitatory postsynaptic potentials (EPSPs). In order to explore the role of CIIN in synaptic transmission in neuronal networks, we addressed the following questions: (i) Is NMDAR-mediated Ca^2+^ influx during unitary synaptic events sufficient to produce detectable CIIN? (ii) Does CIIN depend on membrane potential due to the voltage-dependent Mg^2+^ block of NMDARs? (iii) Does CIIN affect unitary EPSP kinetics and temporal summation of postsynaptic potentials?

We report that manipulating the Ca^2+^ buffer capacity of hippocampal CA1 pyramidal neurons strongly affects the amplitude of single, subthreshold NMDAR-mediated EPSPs. Moreover, upon high-frequency afferent stimulation, simultaneous relief from Mg^2+^ block and CIIN increased the contribution of NMDARs to postsynaptic EPSPs and significantly prolonged their decay time. Our findings suggest that Ca^2+^ flux induced during unitary synaptic events is sufficient to produce detectable inhibition of NMDARs. Repetitive activation of excitatory synapses results in a significant prolongation of the integration window for synaptically evoked action potentials (APs).

## Materials and Methods

All experimental protocols were performed in accordance with the Kazan Federal University regulations on the use of laboratory animals (ethical approval by the Institutional Animal Care and Use Committee of Kazan State Medical University N9–2013) or by the state government of Baden-Württemberg, Germany. All efforts were made to minimize animal suffering and to reduce the number of animals used.

Transverse hippocampal 250 μm slices were prepared from the brains of 14–21 days-old mice (C57 BL/6J), killed by cervical dislocation. The slicing chamber contained an oxygenated ice-cold solution (modified from Dugue et al., 2005) composed of (in mM): K-Gluconate, 140; N-(2-hydroxyethyl) piperazine-N′-ethanesulfonic acid (HEPES), 10; Na-Gluconate, 15; ethylene glycol-bis (2-aminoethyl)-N, N, N′, N′-tetraacetic acid (EGTA), 0.2; and NaCl, 4 (pH 7.2). Slices were incubated for 30 min at 35°C before being stored at room temperature (22–24°C) in artificial CSF (ACSF) containing (in mM): NaCl, 125; NaHCO_3_, 25; KCl, 2.5; NaH_2_PO_4_, 1.25; MgCl_2_, 1; CaCl_2_, 2; and glucose, 25; bubbled with 95% O_2_ and 5% CO_2_. Mg^2+^-free ACSF had 0 mM MgCl_2_ and 0.2 mM EDTA.

Patch electrodes were pulled from hard borosilicate capillary glass (Sutter Instruments flaming/brown micropipette puller). In the experiments conducted in voltage-clamp mode, the intracellular solution consisted of (in mM): Cs-gluconate, 100; CsCl, 40; HEPES, 10; NaCl, 8; MgATP, 4; MgGTP, 0.3; phosphocreatine, 10 (pH 7.3 with CsOH). In the current-clamp experiments, Cs^+^ was substituted for K^+^ in the pipette solution.

Hippocampal CA1 pyramidal cells were identified visually using IR-video microscopy. Whole-cell recordings from these neurons were taken at room temperature using a HEKA EPC-7 amplifier (List Elektronik). To evoke synaptic currents, glass electrodes filled with ACSF were placed in the stratum radiatum within 50–100 μm of the body of the recorded neuron. Inhibitory synaptic transmission was blocked during recordings by the addition of 10 μM gabazine to the perfusion ACSF. The intersweep interval was 6 s. In the voltage-clamp experiments the command voltage was corrected for the liquid junction potential. AMPAR and NMDAR mediated currents were pharmacologically dissected using the AMPAR and NMDAR antagonists, CNQX (10 μM) and APV (50 μM), respectively. After recording the total current responses (containing both AMPAR and NMDAR components, 100 sweeps), AMPAR channels were blocked by bath application of CNQX (10 μM) and another 100 sweeps containing only NMDAR responses were recorded. To confirm that the remaining current after CNQX application was NMDAR-mediated; APV (50 μM) was applied at the end of every experiment. AMPA currents were obtained by subtraction of the averaged NMDA response from the averaged total response. AMPA/NMDA ratios were calculated as the peak AMPAR-mediated current amplitudes divided by the peak NMDAR-mediated current amplitudes.

During recordings, membrane resistance was monitored, and data from cells in which the membrane resistance varied by >15% were discarded from the analysis. Throughout the article *n* refers to the number of the experiments in the group.

For statistical analysis, the Mann-Whitney test has been used and data are presented as mean ± *SD*, unless otherwise stated.

## Results

### Ca^2+^ Entry During Unitary Subthreshold Synaptic Responses is Sufficient to Trigger CIIN

**Figure 1:**
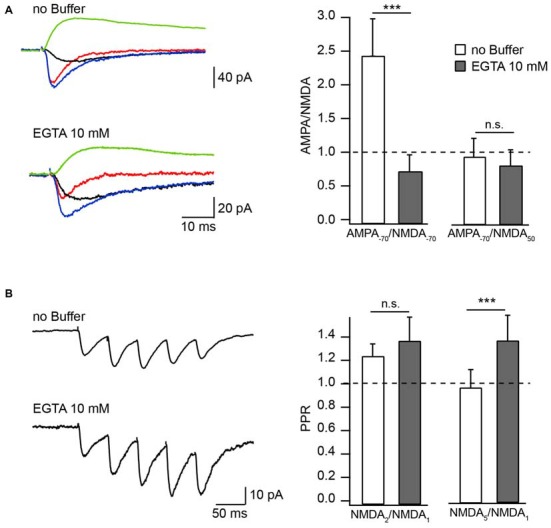
**Effect of Ca^2+^ buffers on the amplitude of NMDAR mediated EPSC in Mg^2+^-free extracellular solution. (A)** Averaged evoked compound EPSC (blue), aEPSC (red), nEPSC (black) at −70 mV and nEPSC (green) at 50 mV recorded with buffer-free and EGTA-containing intracellular solutions. Bar histograms compare the effect of buffer loading on amplitude ratios of aEPSCs measured at −70 mV to nEPSCs measured at −70 mV (left) and 50 mV (right). **(B)** Averaged trains of NMDAR mediated EPSCs recorded with EGTA-free and EGTA-containing solutions. Bar histograms show the effect of EGTA loading on amplitude ratios (PPR) of the second (left) and the fifth (right) nEPSCs to the amplitude of the first response. Asterisks indicate significant difference.

The physiological significance of CIIN depends on two major questions: (i) Is the onset of CIIN fast enough to affect the amplitude of the NMDAR response during a unitary synaptic event? and (ii) Is the concentration of Ca^2+^ entering the spine during subthreshold EPSPs/EPSCs sufficient to trigger CIIN? Depending on these parameters, the effect of CIIN can be either nearly instant, modulating all postsynaptic responses, or can be mostly cumulative, being pronounced during repetitive activity at high frequencies. Manipulation of [Ca^2+^]_i_ by changing the intracellular Ca^2+^ buffer capacity provides a powerful tool to discriminate between those two scenarios. Hippocampal CA1 pyramidal neurons have naturally low endogenous buffer expression (Scheuss et al., 2006). To evaluate the kinetics of CIIN, we examined the effect of intracellular buffer loading (10 mM EGTA) into CA1 pyramidal neurons, on the amplitude of Schaffer-collateral evoked NMDAR-mediated EPSCs (nEPSCs). Experiments were carried out in Mg^2+^-free ASCF. Responses were measured either at −70 mV or at +50 mV and were compared to the amplitude of AMPAR-mediated EPSCs (aEPSCs) recorded from the same cell at −70 mV. At −70 mV, the relative amplitudes of nEPSCs recorded in the presence of EGTA were substantially higher compared to those measured with buffer-free pipette solution (Figure 1A). Accordingly, the AMPA/NMDA ratio recorded with EGTA-containing intracellular solution was 0.7 ± 0.26 (*n* = 6) and under EGTA-free conditions it was 2.44 ± 0.55 (*n* = 7, *p* = 0.001). Ratios of aEPSCs recorded at −70 mV to nEPSCs acquired at +50 mV were still slightly lower in the presence of the buffer, however the apparent difference was not significant (EGTA-free 0.94 ± 0.28, *n* = 7); EGTA-containing 0.81 ± 0.24 (*n* = 6; *p* = 0.45; Figure 1A). These data show that at −70 mV EGTA loaded into the cell prevents CIIN by buffering incoming Ca^2+^, resulting in increased nEPSC amplitudes compared to those in buffer-free conditions. Whereas at +50 mV, when Ca^2+^ entry is negligible, the nEPSC amplitude is practically insensitive to buffer loading due to lack of CIIN. The latter also indicates that buffer loading does not trigger any long lasting voltage-independent change in synaptic NMDAR conductance.

To test whether the degree of CIIN can be increased by prolonged subthreshold synaptic stimulation, we measured and compared the amplitude ratios of the second (NMDA2) and fifth (NMDA5) nEPSCs to the first nEPSC (NMDA1), using a five-pulse stimulation protocol (10 Hz) in neurons loaded with buffer-free or EGTA-containing intracellular solutions (Figure 1B). The averaged NMDA2/NMDA1 ratio was increased slightly, but not significantly, by buffer loading (EGTA-free: 1.23 ± 0.11, *n* = 6; EGTA-containing: 1.36 ± 0.21, *n* = 5; *p* = 0.662). Later responses, however, were clearly enhanced by buffering Ca^2+^: the NMDA5/NMDA1 ratio was 0.9 ± 0.15 in neurons patched with EGTA-free solution (*n* = 6) and 1.28 ± 0.21 in EGTA-containing neurons (*n* = 5; *p* = 0.009). These data indicate that CIIN does alter the NMDAR contribution to unitary responses, with a stronger impact on NMDAR-mediated currents during prolonged repetitive activity.

### In the Presence of Mg^2+^ CIIN Affects NMDAR-Mediated Currents in a Voltage-Independent Manner

**Figure 2:**
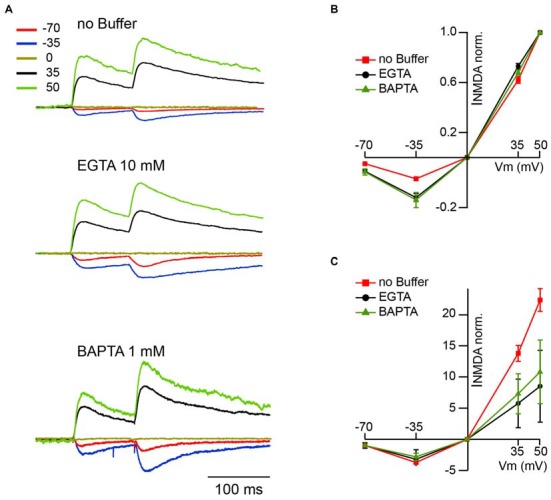
**CIIN strongly reduces the amplitudes of nEPSCs at negative potentials, but does not change the voltage dependence of the Mg^2+^ block. (A)** Normalized (50 mV) synaptically evoked NMDAR-mediated responses recorded at −70, −35, 0, 35 and 50 mV from CA1 pyramidal cells dialyzed with buffer-free, EGTA- or BAPTA-containing intracellular solutions. **(B,C)** Current voltage relationships of nEPSCs normalized to the amplitudes at 50 mV **(B)** and −70 mV **(C)** obtained with buffer-free (red), EGTA- (black) and BAPTA-containing (green) intracellular solutions.

The next question was whether under physiological conditions, when NMDARs are heavily controlled by extracellular Mg^2+^, CIIN could still influence nEPSCs? The magnitude of the Mg^2+^ block at resting membrane potentials is nearly maximal, resulting in a robust reduction of the NMDAR contribution to postsynaptic Ca^2+^ influx. On the contrary, at less negative potentials, upon partial relief of the block, enhanced Ca^2+^ influx through the channels might still have significant consequences on NMDAR function. In other words, the nonlinearity of NMDAR-mediated Ca^2+^ entry due to Mg^2+^ block could give rise to a voltage dependence of CIIN. To test this hypothesis, we investigated the effect of intracellular buffer loading on current voltage (IV) relationships of evoked synaptic NMDAR-mediated currents. Figure 2A shows averaged evoked nEPSCs measured at −70, −35, 0, 35 and 50 mV with buffer-free, EGTA-containing (10 mM) and BAPTA-containing (1 mM) intracellular solutions. All responses were normalized to the mean EPSC amplitude obtained at 50 mV, where reduced Ca^2+^ entry through the channels should have a minor effect on the amplitudes of nEPSCs. Normalized nEPSCs measured at 35 mV were nearly identical, irrespective of the intracellular buffer content. However, responses recorded at −35 and −70 mV with EGTA (nEPSC_−35_: −0.32 ± 0.07; nEPSC_−70_: −0.11 ± 0.03; *n* = 6) or BAPTA (nEPSC_−35_: −0.34 ± 0.09; nEPSC_−70_: −0.12 ± 0.03; *n* = 5) in the patch pipettes were more than twofold larger than those collected with buffer-free intracellular solution (nEPSC_−35_: −0.17 ± 0.04; nEPSC_−70_: −0.05 ± 0.01; *n* = 7; Figure 2B). The enhancement of NMDA-mediated EPSCs in the presence of intracellular Ca^2+^ buffers was highly significant at both negative recording potentials (−35 mV, p ≪ 0.001; −70 mV, *p* < 0.001 one way ANOVA). However, after normalization to the values obtained at −70 mV, the normalized nEPSC_−35_ amplitudes in all three groups were not different (−3.71 ± 0.5, −2.72 ± 0.6 and −3.2 ± 0.9 for buffer-free, EGTA- and BAPTA-containing solutions respectively; *p* > 0.05 one way ANOVA; Figure 2C), indicating that the magnitude of CIIN did not depend on the strength of the Mg^2+^ block. Thus, CIIN can drastically reduce nEPSC amplitudes even in the presence of Mg^2+^ at physiological concentrations.

### The Relative Contribution of NMDAR to the Postsynaptic Responses is Strongly Controlled by CIIN

**Figure 3:**
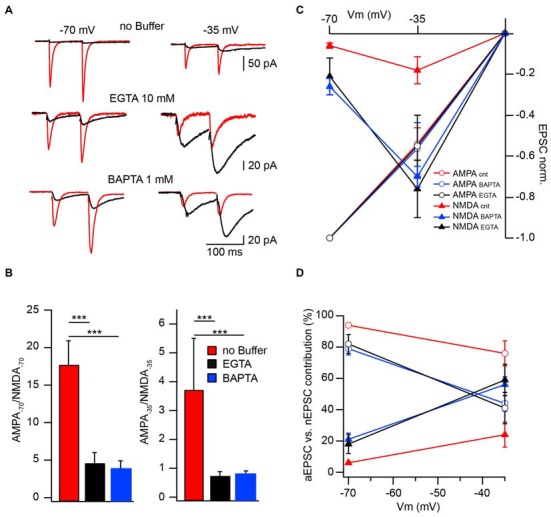
**Effect of CIIN on NMDAR contribution to compound EPSCs. (A)** AMPAR- (red) and NMDAR- (black) mediated responses recorded at −70 and −35 mV from neurons with buffer-free or buffer-containing pipette solution. **(B)** Bar histograms compare the effects of EGTA or BAPTA loading on AMPA/NMDA ratios obtained at −70 (left) and −35 mV (right). **(C)** Weighted IVs of synaptic aEPSCs (circles) and nEPSCs (triangles) measured from neurons dialyzed with buffer-free (red), EGTA- (black) or BAPTA –containing (blue) intracellular solutions. **(D)** Voltage dependence of the relative NMDAR-contribution to the synaptic EPSC in the presence or absence of Ca^2+^ buffers. Labeling is the same as on **(C)**. Asterisks indicate significant difference.

To further substantiate the modulatory role of CIIN under physiological conditions and estimate its impact on the amplitude of compound EPSCs we compared AMPAR- and NMDAR-mediated responses measured at −70 and −35 mV in neurons patched with buffer-free and buffer-containing pipette solutions. At both holding potentials, the relative amplitudes of nEPSCs recorded from the cells dialyzed with buffer-free intracellular solution were significantly smaller compared to those measured with EGTA (10 mM) or BAPTA (1 mM), as indicated by much smaller AMPA/NMDA ratios (Figures 3A,B).

To evaluate the effect of CIIN on the relative NMDAR contribution to the compound response, we reconstructed weighted synaptic IV-curves of aEPSCs and nEPSCs recorded with buffer-free and buffer-containing solutions. Both AMPAR- and NMDAR-mediated responses were normalized to the averaged aEPSC amplitude measured at −70 mV (Figure 3C). As expected aEPSC amplitudes did not depend on the intracellular buffer content and the IVs of the aEPCSs were nearly linear. However, the weight of the NMDAR contribution to the compound EPSCs strongly depended on the presence of Ca^2+^ buffers. In the cells dialyzed with buffer-free solution at −70 mV the contribution of the nEPSC was 6 ± 1% of the compound response. The impact of NMDARs increased at −35 mV (24 ± 8%) but was still significantly lower than that of AMPARs (*n* = 8; *p* < 0.001; Figure 3D). In neurons loaded with buffers, the contribution of NMDAR channels was strongly enhanced at −70 mV (10 mM EGTA: 18 ± 6%, *n* = 6; 1 mM BAPTA 21 ± 4%, *n* = 5), moreover, at −35 mV weighted nEPSC amplitudes were significantly larger than aEPSCs (59 ± 10% and 56 ± 5%, *p* < 0.05 for EGTA- and BAPTA-containing solutions respectively). Thus relief from CIIN gave rise to a three to fourfold enhancement in NMDAR contribution to excitatory postsynaptic responses.

### CIIN Moderates the EPSP Decay Time and Action Potential Firing Window

**Figure 4:**
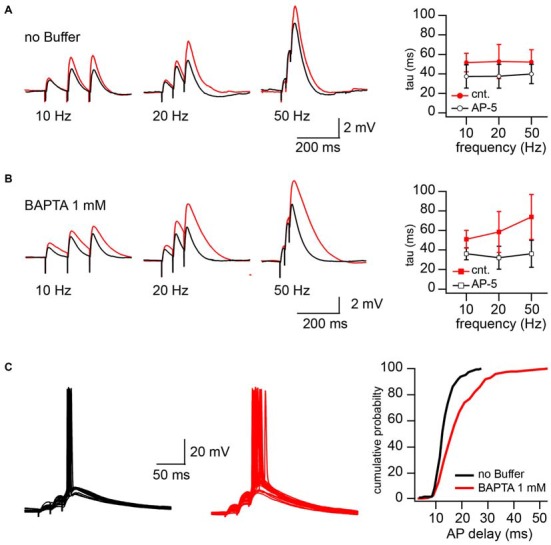
**CIIN significantly accelerates decay of EPSPs and shortens the time window for action potential (AP) generation. (A)** EPSPs evoked by 10, 20, and 50 Hz trains in cells patched with buffer-free intracellular solution in the presence (black) and absence (red) of 50 μM APV. The plot shows the effect of NMDAR blockade on the decay time constant of the last response at different stimulation frequencies. **(B)** The same as in **(A)**, obtained from the neurons loaded with 1 mM BAPTA. **(C)** Synaptically evoked APs recorded with buffer-free (black) and BAPTA-containing (red) pipette solutions. The plot represents the dependence of AP delays (latencies) on the cellular buffer content.

The increased NMDAR contribution in CIIN-free conditions may substantially prolong EPSP duration and as a result, increase the time window for AP generation. To test these possibilities, we examined the consequences of intracellular BAPTA (1 mM) loading on the EPSP decay time constant and neuronal firing properties. Experiments were carried out in current clamp mode, and postsynaptic CA1 pyramidal cells were kept at resting membrane potential (around −65 mV). To reach different levels of postsynaptic depolarization, Schaffer collateral inputs were stimulated with 50, 20 and 10 Hz trains of three stimuli. The stimulation intensity was the minimal and sufficient intensity to trigger, with 40–60% probability, an AP in response to the 3rd stimulus in 50 Hz trains. Reduction of the stimulation frequency to 20 and 10 Hz decreased both contribution of the EPSPs temporal summation and paired-pulse facilitation to the peak depolarization reached by the 3rd EPSP. After recording 50–75 sweeps at each stimulation frequency, NMDARs were blocked by bath application of the receptor antagonist APV (50 μM) and additional 50 responses were collected at 50, 20 and 10 Hz, respectively. To estimate the contribution of NMDARs to the EPSP duration we compared the decay time constants (tau) of the averaged 3rd EPSPs before and after APV application. Sweeps with APs (occurring at 50 Hz stimulation) were excluded from EPSP decay analysis. In cells patched with buffer-free intracellular solution APV application caused a small, but non-significant acceleration of EPSP decay. The change in the EPSP time constant did not depend on stimulation frequency, the averaged tau values were (in milliseconds) 52 ± 12 vs. 38.9 ± 10 (50 Hz), 52.7 ± 17.5 vs. 37.6 ± 12.2 (20 Hz) and 51.6 ± 9.5 vs. 37.4 ± 12 (10 Hz) before and after drug application respectively (*n* = 5; *p* > 0.05; Figure 4A). However, in the BAPTA loaded neurons, activation of synaptic NMDARs had a drastic effect on EPSP decay. At 50 Hz tau values were, in control 74 ± 23, and decreased to 36 ± 14 ms in the presence of APV (*n* = 5; *p* = 0.008; Figure 4B). At 20 Hz the acceleration of EPSP decay constants by APV was still significant (58.4 ± 21 vs. 34 ± 12 ms; *p* = 0.029), while at 10 Hz, block of NMDARs did not cause a meaningful tau reduction (51 ± 9.2 vs. 36.1 ± 6.24 ms; *p* = 0.2). Note that the time constants measured in the presence of APV were very similar to those in the neurons patched with buffer-free and BAPTA-containing solutions.

In line with the prolongation of EPSP decay, AP delays (latency), measured as the interval between the 3rd stimulus artifact and the peak of the AP, were significantly longer in the BAPTA loaded neurons. Figure 4C shows superimposed traces recorded from neurons recorded with buffer-free (black) and BAPTA-containing (red) pipette solutions. Cumulative probability plots (right) show the shift towards longer AP delays in the presence of BAPTA (pooled data from five cells in each group; *p* < 0.001, Kolmogorov–Smirnov test). Thus, ablation of CIIN and consequent enhancement of the NMDAR contribution to the EPSP duration considerably prolonged the window for AP generation.

## Discussion

It is generally accepted that the main functional role of NMDARs is related to their high permeability to Ca^2+^, which confers on NMDARs a central role in both synaptic plasticity and neuronal survival under physiological conditions and neuronal death under excitotoxic pathological conditions (Paoletti et al., 2013).

Functional consequences of NMDARs modulation by various signaling molecules and biochemical cascades under physiological conditions were extensively studied over the last two decades. However the functional role of CIIN remains poorly understood.

### CIIN as a Mechanism of NMDARs Self-Regulation Under Physiological Conditions

The phenomenon of Ca^2+^ induced inhibition of NMDARs, has been well documented and explored at the level of intracellular molecular mechanisms (Legendre et al., 1993; Medina et al., 1994; Rosenmund et al., 1995; Ehlers et al., 1996; Wang and Wang, 2012; Paoletti et al., 2013; Bajaj et al., 2014; Yang et al., 2014). However, an important question remained open, namely whether the Ca^2+^ entry associated with single NMDAR mediated synaptic events under physiological conditions is sufficient to self-inhibit NMDAR mediated responses. In other words, do the mechanisms governing CIIN operate on the EPSCs time scale (milliseconds)?

These aspects of CIIN have not been addressed in previous studies, where CIIN was triggered either by Ca^2+^ entry through voltage gated calcium channels or by the prolonged activation of NMDARs (Medina et al., 1994, 1995, 1996). These studies also did not strictly quantify the magnitude of NMDAR “self-inhibition”, especially under physiological conditions. However, Ehlers et al. (1996) provided convincing evidence that 50 μM [Ca^2+^]_i_ in the presence of calmodulin causes a ~4 fold reduction of open probability and shortens channel open times of NMDARs by half.

We have found that Ca^2+^ entering trough NMDAR during a unitary synaptic event can strongly attenuate the amplitude of nEPSCs indicating that the CIIN operates on a rapid time scale of a few milliseconds. This data is in agreement with our previous findings on recombinant channels where in outside out patches, Ca^2+^ influx triggered by brief (1 ms) activation of Ca^2+^ permeable AMPARs was sufficient to reduce current amplitude of a co-expressed and co-activated Ca^2+^-impermeable NMDAR mutant (Rozov et al., 1997). Moreover, in physiological Mg^2+^ concentrations, even around resting membrane potential, where the strength of the Mg^2+^ block is nearly maximal, NMDARs can still conduct a sufficient amount of Ca^2+^, to produce a nearly fourfold reduction in the channel’s function. Indeed, according to Kovalchuk et al. (2000) under these conditions, subthreshold afferent stimulation gives rise to detectable [Ca^2+^]_i_ in the spines of CA1 pyramidal cells which is almost exclusively mediated by NMDARs. Together with the fact that Ca^2+^ influx through NMDARs is detectable up to at least +20 to +40 mV (Burnashev et al., 1995; Kovalchuk et al., 2000) this suggests that CIIN is operative under physiological conditions.

Our data strongly suggest that this elevation in [Ca^2+^]_i_ is sufficient to trigger CIIN and onset of the inhibition is fast enough to shape individual postsynaptic responses. This finding is in perfect agreement with data on the magnitude of CIIN on the single channel level (Ehlers et al., 1996). Thus, we provide the first evidence that under physiological conditions synaptic NMDARs in cells with low buffer capacity are drastically self-inhibited by NMDAR-mediated Ca^2+^ influx.

However, in the vast majority of GABAergic interneurons the contribution of NMDARs to the Ca^2+^ homeostasis is strongly moderated by an increased endogenous buffer capacity (Freund and Buzsáki, 1996). On the other hand, the presence of endogenous buffers like parvalbumin (PV), calretinin (CR) or calbindin (CB) can effectively reduce the magnitude of CIIN, increasing the contribution of NMDARs to the postsynaptic response. Indeed, synaptic expression of the GluN1 subunit in the hippocampal PV positive interneurons is several fold lower than that in CA1 pyramidal cells (Nyiri et al., 2003), nevertheless, the values of AMPA/NMDA ratios measured in these neurons are very similar (Fuchs et al., 2007). Thus, relief from CIIN by endogenous Ca^2+^ buffers changes the main job of NMDARs from the synaptic Ca^2+^ supplier to the active postsynaptic contributor to the amplitude and decay of EPSPs. Functionally this can minimize the role of NMDAR channels in the induction of long-term plasticity, but increase their impact on EPSP temporal summation and AP firing profile (Figure 4). It has been found recently that NMDA spikes occurred in multiple dendritic branches of layer 2/3 pyramidal neurons both spontaneously and as a result of sensory input have a major role in enhancing neuronal output in neocortex (Palmer et al., 2014). Contribution of CIIN to the local NMDA spikes, therefore, may also have influence on the number of output APs thus affecting sensory processing and network activity in the cortex.

### Possible Role of CIIN During Aging and in Neuropsychiatric Disorders

Change of NMDARs function have also been implicated in the development of psychotic symptoms in the number of neuropsychiatric illnesses (Lakhan et al., 2013). Along with this the expression of endogenous Ca^2+^ buffers changes during aging and some neurological disorders (Bu et al., 2003; Riascos et al., 2011). Thus, the selective vulnerability of the basal forebrain cholinergic neurons to degeneration in Alzheimer’s disease has been attributed to the age-related loss of CB from these neurons and a consequent rise in intracellular Ca^2+^ (Riascos et al., 2011). Under these conditions CIIN may play an intrinsic compensatory role counteracting intracellular Ca^2+^ elevation by reducing NMDAR-mediated Ca^2+^ entry.

Interestingly, alteration in expression of Ca^2+^ buffers often aligns with the alteration of NMDAR function. For instance, age-dependent reduction of CR expression in hippocampal granular cells coincides with down regulation of GluN1 immunoreactivity (Gazzaley et al., 1996). Finally, a number of neurological disorders are associated with dysregulation of both NMDAR function and endogenous Ca^2+^ buffer synthesis (Heizmann and Braun, 1992; Paoletti et al., 2013; Kook et al., 2014). It has been suggested that PV alterations in schizophrenia can consequently lead to the hypofunction of NMDARs. Schizophrenia is often attributable to NMDAR hypofunction, this might reflect dysregulation of the receptor rather than a deficit in the number of NMDARs (Kantrowitz and Javitt, 2010; Gonzalez-Burgos and Lewis, 2012). In addition, the variation in extracellular Ca^2+^ concentration under certain conditions may also attenuate the impact of the CIIN. For example, *in vivo* measurements of extracellular Ca^2+^ concentration in primates during seizures has shown that the Ca^2+^ level drops to within the 100 μM range (Pumain et al., 1985). In this case reduced CIIN may increase the window for synaptic integration due to the prolongation of the NMDAR mediated response and lead to neuronal overexcitability.

In conclusion, our findings suggest that Ca^2+^ induced inactivation of NMDARs operating on the time scale of EPSCs may contribute to the cell-specific fine tuning of excitatory synaptic transmission under normal and pathological conditions.

## Author Contributions

Study conception and design: AD, NB and AR. Acquisition of the data: FV, YZ and AR. Analysis and interpretation of the data: FV and AR. Analysis of the data: MM. Drafting of manuscript: AD, NB and AR. Critical revision: NB and AR.

## Conflict of Interest Statement

The authors declare that the research was conducted in the absence of any commercial or financial relationships that could be construed as a potential conflict of interest. The Review Editor Dr. Cinzia Costa declares that, despite being affiliated with the same institution as the Associate Editor, Dr. Maria Cristina D’Adamo, the review process was handled objectively.
